# Traumatism and Disease: Difficulties under the Workmen's Compensation Act

**Published:** 1915-05-29

**Authors:** 


### TRAUMATISM AND DISEASE.

### Difficulties Under the Workmen's Compensation Act.

In dealing with claims which arise under the Workmen's Compensation Act or other insurance scheme, there is one class of case in which the difficulty of giving a confident medical opinion is extreme. We refer to those instances in which shortly after the accident there appear evidences of disease. The question immediately arises, Is the violence which the patient has suffered the "cause" of the disease? To the lawyers this seems a very simple and direct issue, and, as is their wont, they prescribe two answers as alone possible—namely, yes and no. To the medical practitioner, however, the matter is far from simple; it is, indeed, very complex. In the majority of cases the disease in question occurs in circumstances from which all history of traumatism is absent. But does this necessarily mean that in the individual case violence cannot have been the causal agency? Even if it is allowed that other agencies than trauma have played some part in the chain of causation, does this prove that the violence suffered has played none? Is it or is it not possible to say that the patient would certainly have developed the disease had he not been injured? These are questions which press with great anxiety on the conscientious medical adviser, and they are the more anxious seeing that his decision one way or the other occupies a position of commanding importance. It is by what he says that the action of the lawyer and his client will be determined. The pressure of both of these is towards an absolute and unqualified reply. Such a reply, in many circumstances, is quite impossible, and we wish to suggest that to this position it is the duty of the medical witness to adhere. To the legal mind this proceeding may be unsatisfactory. But with this we are not concerned. To go in advance of the facts as we know them is impossible.

The general principle to be borne in mind by a medical witness concerned in such a discussion as is here in issue is that there is a danger of unduly limiting the part which may be played by violence as a cause of disease. He sees many cases of a similar order in which there is no suspicion of injury as a factor in their development, and the natural inclination is to say, when violence is an event in the recent history, that the conjunction of the two is a mere coincidence. There are many records which ought to abate this confidence. Take, for example, the case of phthisis pulmonalis. Every practitioner sees scores of such cases in which no suspicion of the influence of injury is even suggested. But now and again a person believed to be in good health is subjected to some form of violence, and promptly thereafter evidences of pulmonary tubercle appear. The connection between the two may be difficult to explain, but to say that there is not, and cannot be, any connection at all is surely a rash position to adopt. In a legal argument it may be urged that the cause of pulmonary tuberculosis is the tubercle bacillus, and probably no one will be found to contest this proposition. But do we know all the conditions which determine the activity of the bacillus, and are we sure that the state of the tissues induced by violence is not one of them? To answer these questions confidently, either in the affirmative or in the negative, needs some courage, and we doubt whether either the one or the other position can claim scientific justification. Probably in this particular instance there are sufficient clinical experiences to incline the balance of opinion in favour of the view that traumatism may in a practical sense be responsible for the appearance of active evidences of tubercle, but the doctrine can hardly be stated as an absolute one. Certainly to deny the doctrine as incredible and impossible cannot claim justification. But if it be allowed that records showing the occasional development of tuberculous disease shortly after an injury justify the suggestion that the relation between the two may be a causal one, the admission has a far-reaching influence. Similar experiences may be quoted in other conditions, as, for example, in diabetes, in cancer, and in various forms of nervous disease. In face of such facts how is it possible confidently and absolutely to limit the *rôle* which may be played by violence as a cause of disease in an individual patient?

A case recently tried in one of the Courts will illustrate the point we wish to make. It was admitted that the plaintiff had suffered considerable violence in a cab accident, and he allowed that from the grosser consequences of this he had recovered. But he alleged that since the accident he had, even on moderate exertion, been subjected to breathlessness and palpitation, and that these symptoms interfered both with his comfort and with his capacity to carry on his business. The medical witnesses both on one side and the other agreed that the man was suffering from degenerative changes in his cardiac muscle, but while those called by the plaintiff affirmed that such changes might possibly be determined by violence, the defendant's witnesses maintained that they were the result of disease, and that between them and the accident which the plaintiff had suffered there was no possible connection. With the merits of the individual case we are not concerned. But we should like to know the reasons which in the minds of the defendant's witnesses justified the sweeping proposition that violence cannot possibly exercise causative influence in the production of cardiac degeneration. To say that such degeneration is often seen in practice apart from violence is very far from closing the debate. Individual experiences may justify individual opinions, but cannot limit possibilities. Our point, however, is not merely to emphasise the necessity of dealing very cautiously with cases in which symptoms of disease follow the application of violence, and especially the need for avoiding a hurried opinion that because not usually associated the two have no relation.

